# Primary EWS/PNET of the lung with TP53 germline and SKT11 somatic mutation: A case report and review of the literature

**DOI:** 10.1111/1759-7714.14251

**Published:** 2021-12-05

**Authors:** Shuangping Zhang, Yun Chen, Shiping Guo, Ke‐Neng Chen

**Affiliations:** ^1^ Department of Thoracic Surgery Shanxi Cancer Hospital Taiyuan China; ^2^ Department of Operating Room Nursing Shanxi Cancer Hospital Taiyuan China; ^3^ The First Department of Thoracic Surgery Peking University Cancer Hospital & Institute Beijing China

**Keywords:** Ewing's sarcoma, pulmonary, TP53

## Abstract

Primary pulmonary EWS/PNET is extremely rare. This report describes a 20 year‐old man with primary pulmonary EWS/PNET with TP53 germline and SKT11 somatic mutation. After four neoadjuvant chemotherapy cycles (VAC with alternating IE) combined with anlotinib, a left pneumonectomy was performed. Maintenance anlotinib monotherapy was then continued with no sign of recurrence to date. It is suggested that before the treatment and prognosis of children or young adults with primary EWS/PNET of the lung that consideration should be given to genetic testing.

## Graphical abstract

Primary pulmonary EWS/PNET is extremely rare. This report describes a 20 year‐old man with Primary pulmonary EWS/PNET with TP53 germline and SKT11 somatic mutation.After 4 neoadjuvant therapy, R0 resection was obtained. Then maintenance anlotinib monotherapy was continued, no sign of recurrence to date as an outcome.

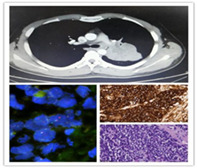

## INTRODUCTION

Ewing's sarcoma is a rare and highly invasive mesenchymal tumor which belongs to the neuroectodermal tumor family, and includes Ewing's osteosarcoma, extra‐skeletal Ewing's sarcoma, primitive neuroectodermal tumor (PNET), Askin tumor and atypical Ewing's sarcoma.^1^, ^2^ It accounts for 6%–8% of primary bone tumors in children and young adults, and its incidence peaks at 20, mostly in males.^3^ Extra‐skeletal EWS/PNET is clinically rare with very low incidence, which accounts for about 1% of soft tissue sarcoma.^2^ Primary pulmonary EWS/PNET (PPES) is extremely rare. Since Hammar et al. reported the first case of PPES in 1989,^4^ less than 40 cases have been reported in the literature.^5^ Here, we discuss the clinical course and treatment in one case of PPES with TP53 germline and SKT11 somatic mutation.

## CASE REPORT

**FIGURE 1:**
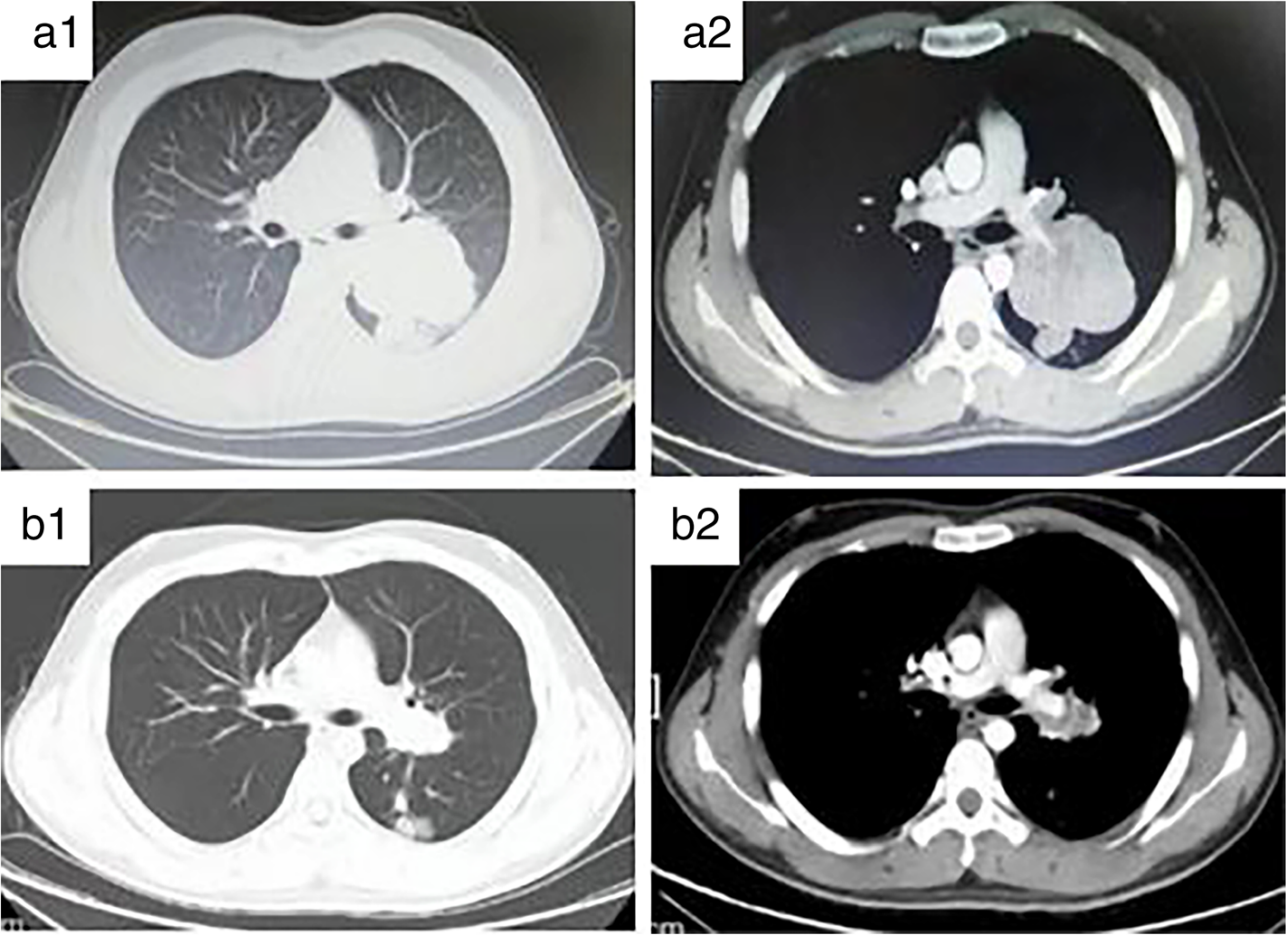
CT thorax. (a1) A large irregular and well‐defined border mass lesion measuring 7.7 × 6.3 cm beside the left hilus pulmonis and an enlarged mediastinal lymph node measuring 2.7 × 1.9 cm. (a2) The left inferior pulmonary artery is surrounded and the left pulmonary artery was squeezed and deformed. (b1) The tumor was remarkably reduced to 2.5*3.4 cm and the mediastinal lymph node was 1.2 cm. (b2) The left pulmonary artery was surrounded, which was slightly narrower

**FIGURE 2:**
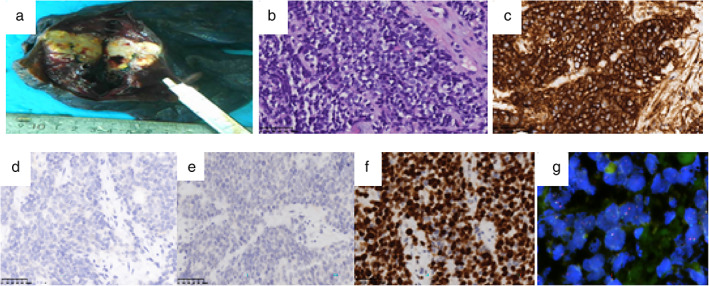
Pathological examination. (a) The cut surface of the mass in the left pneumonectomy specimen shows 3.5 × 3 × 3 cm sized white‐gray and yellow‐gray areas with sharp demarcation. (b) H&E × 400 the proliferation of small round tumor cells. (c) CD99(+) × 400. (d) AE1/AE3(−) × 400. (e) TTF‐1(−) × 400. (f) Ki67(approximately 80% + ×400). (g) FISH shows EWSR1 gene rearrangement

**FIGURE 3:**
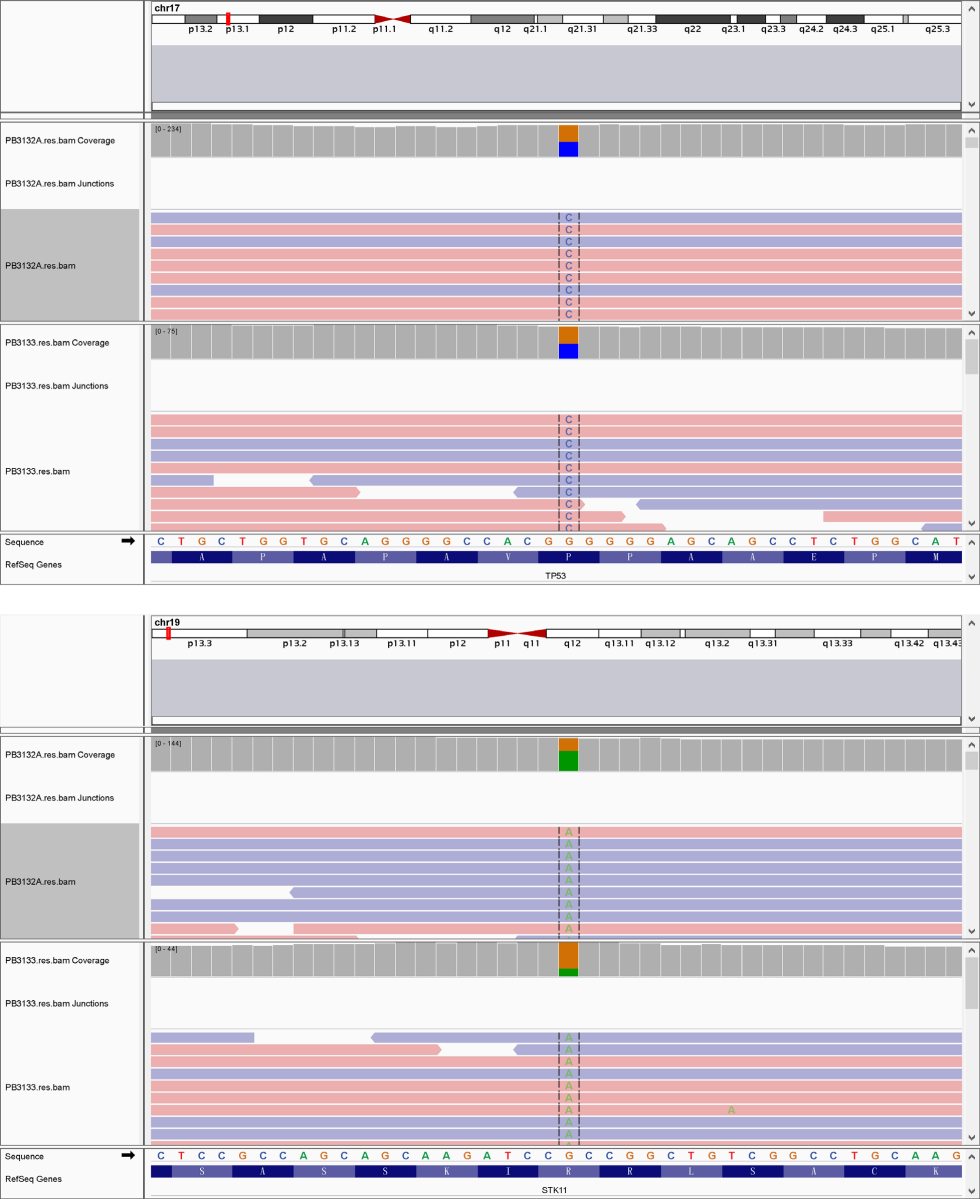
Whole exome sequencing (WES). TP53 germline mutation: NM_000546:exon4:c.C215G:p.P72R; SKT11 somatic mutation: NM_000455:exon9:c.G1274A:p.R425H

A 20 year old man was admitted to our department with a history of a cough for 20 days. His family history was that his grandfather had suffered from lung cancer. A CT scan of thoracic showed a large irregular and well‐defined border mass lesion measuring 7.7 × 6.3 cm with inhomogenous enhancement next to the left hilus pulmonis across the interlobar fissure and an enlarged mediastinal lymph node measuring 2.7 × 1.9 cm (Figure 1a1,a2). No intrabronchial lesion was found on bronchoscopy. A CT‐guided needle biopsy of the left lung lesion was performed which revealed that small and round cells were distributed in nests microscopically, with abnormal nuclei. AE1/AE3 (−), CD99 (+), NSE (−), Ki67 (approximately 80%+), TTF‐1 (−), Syn (+), NKX2.2 (−),CD56 (+), and CgA (−) was reported by immunohistochemistry (IHC). A fluorescent in situ hybridization (FISH) assay showed a positive rearrangement of the ESWR1 gene (Figure 2). Thus, a definitive diagnosis of PPES was made. The neoadjuvant chemotherapy regimen consisted of four alternating cycles of VAC (vincristine, 1.5 mg/m^2^, intravenous glucose tolerance test [ivgtt], D1, D8 and D15; actinomycin D 1.5 mg/m^2^, ivgtt, D1; cyclophosphamide, ivgtt, 1.2 g/m^2^, D1) and IE (ifosfamide 1.8 g/m^2^, ivgtt, D1–D5; etoposide 100 mg/m^2^, ivgtt, D1–D 5) repeated every three weeks. The first three cycles were combined with anlotinib (12 mg, per os, q.d., D1–14 every 3 weeks). After four cycles of neoadjuvant chemotherapy, the left mass had significantly reduced to 3.4x 2.5 cm on thoracic CT re‐examination (Figure 1b1,b2). A left total pneumonectomy was performed, and postoperative pathological results showed that tumor cells in the left lung were small and round distributed in nests microscopically and pathological mitosis could be seen. Degeneration of the tumor cells, proliferation of interstitial fibrous tissue, and inflammatory cell infiltration conforming to the changes seen after treatment were evident. Metastasis of a subcarinal lymph node was confirmed by pathology. A germline TP53 mutation and SKT11 somatic mutation (Figure 3) was confirmed by whole exome sequencing. Maintenance anlotinib (12 mg, per os, D1–14, every 3 weeks) monotherapy was continued, without disease progression for five months after surgery.

## DISCUSSION

PPES is rare, and the clinical symptoms of cough, phlegm, chest pain, hemoptysis or fatigue, etc are nonspecific. The disease has been found to occur at any age, mostly in children and adolescents, and peaks at the age of 20–30 years old, without any difference in gender.^5^, ^6^, ^7^

CT characterization of PPES is still limited^8^, ^9^, ^10^ and the masses are usually described as being of different size, single or multiple, with clear boundaries and possible lobulation. A mass in a single lung is more common, but can also be seen in both lungs. The mass grows rapidly, may possibly surround the pulmonary artery or vein, or even infiltrate across the pulmonary lobe along the interlobar fissure, and lymph node metastasis was reported in 12% of cases. Upon enhancement, uniform, focal nonuniform or annular enhancement may be observed, and necrosis or hemorrhage can result in low or high density lesions.

Positron emission‐computed tomography (PET‐CT) of PPES often presents with a high SUV value, smooth margins, and high fluorodeoxyglucose uptake. In addition, PET‐CT can help to rule out the possibility of pulmonary metastasis by extra‐pulmonary Ewing's sarcoma.^6^, ^8^, ^11^, ^12^

On light microscopy,^9^, ^10^, ^11^, ^12^ PPES is consisted of small, round or oval shaped cells, with a prominent nucleus, scant cytoplasm and granular chromatin. Homer Wright or Flexner Weinsteiner's rosettes structure could be seen. Necrosis, hemorrhage, and cystic changes are common. Electron microscopy features include high nucleocytoplasmic ratio and aggregation of glycogenosome in cytoplasm. IHC showed characteristic expression of neural differentiation, with high expression of CD99, NSE, FLI‐1 and Syn as relatively specific indicators for diagnosis. Such type of fusion gene is often used to diagnose pathological states, quantitative polymerase chain reaction (qPCR), EWS FLI1 and FLI1 EWS quantitative expression, to reveal the EWSR1 rearrangement by FISH.^3^, ^7^, ^11^, ^12^

There is currently no standard treatment for PPES. Treatment includes surgery, chemotherapy and radiation. Standard first‐line treatment consists of alternating chemotherapy with vincristine, doxorubicin, cyclophosphamide, ifosfamide, and etoposide. Chemotherapy has previously been reported to improve patient survival rate following radical surgery.^13^ The patient reported here opted for neoadjuvant therapy and N2 lymph node metastasis occurred, how to proceed with the next treatment, chemotherapy, or radiation therapy? The patient refused chemotherapy after surgery because of obvious myelosuppression due to the side‐effects caused by chemotherapy. Considering the TP53 gene mutation, the germline mutation of the TP53 gene has only previously been detected in 10% of Ewing's sarcoma patients,^14^ and those patients were younger, with axial tumor location and a poor prognosis.^15^ The incidence of secondary primary tumors in germline TP53 mutation carriers is very high, possibly more than 40%. Studies have found that chemotherapy and/or radiation therapy for the first primary tumor can affect, or cause, secondary primary tumors in these carriers.^16^ Therefore, TP53 testing is necessary, and if possible, radiotherapy and genotoxic chemotherapy should be avoided in carriers. Recent studies have shown that tumor angiogenesis can promote the growth and metastasis of solid tumors, and anlotinib and pazopanib have been reported to be effective in the treatment of PPES, but they are not considered standard treatment.^6^, ^8^ Anlotinib was selected for subsequent treatment after multidisciplinary discussion. To date, five months after surgery, no signs of recurrence were evident in this patient. However, the duration of treatment effect of anlotinib has not previously been reported, and long‐term follow‐up will therefore be required.

## CONFLICT OF INTEREST

The authors report no conflict of interest.
